# Comparative Analysis of Tolerance to Salt Stress and Water Deficit in Two Invasive Weeds of the Genus *Erigeron* (Asteraceae)

**DOI:** 10.3390/plants11152059

**Published:** 2022-08-06

**Authors:** Manel Bellache, Natalia Torres-Pagan, Mercedes Verdeguer, Josep V. Llinares, Leila Allal Benfekih, Radu E. Sestras, Oscar Vicente, Adriana F. Sestras, Monica Boscaiu

**Affiliations:** 1 Mediterranean Agroforestry Institute, Universitat Politècnica de València, Camino de Vera s/n, 46022 Valencia, Spain; 2 Laboratory for Research on Medicinal and Aromatic Plants, Faculty of Nature and Life Sciences, Department of Biotechnology, Université Saad Dahlab de Blida 1, Blida 09000, Algeria; 3 Department of Horticulture and Landscape, University of Agricultural Sciences and Veterinary Medicine of Cluj-Napoca, 400372 Cluj-Napoca, Romania; 4 Institute for the Conservation and Improvement of Valencian Agrodiversity, Universitat Politècnica de València, Camino de Vera s/n, 46022 Valencia, Spain; 5 Department of Forestry, University of Agricultural Sciences and Veterinary Medicine of Cluj-Napoca, 400372 Cluj-Napoca, Romania

**Keywords:** weeds, salinity, water deficit, germination, growth parameters, proline, antioxidants

## Abstract

*Erigeron bonariensis* and *E. sumatrensis* are two noxious weeds present in many parts of the world. Their tolerance to salinity and water deficit was analysed at the seed germination stage and during vegetative development. Seed germination was tested in solutions with different concentrations of NaCl and polyethylene glycol (PEG). Growth parameters, photosynthetic pigments, ion accumulation, and antioxidant mechanisms were analysed in plants that were subjected to increasing NaCl solutions, or severe water deficit by completely restricting irrigation. Seed germination was mostly affected by NaCl, but less by PEG in both species. *E. bonariensis* had a faster germination in all treatments and maintained a higher percentage of germination under the highest concentration of salt applied. Growth responses were similar in the two species, both being more affected by higher salt concentrations than by water deficit. The main differences in the responses of the two species to stress regard K^+^ and proline concentration. K^+^ in roots decreased under salt stress in *E. sumatrensis*, but remained constant in leaves, whereas in *E. bonariensis* increased in roots and leaves in salt-stressed plants. Proline concentration increased in all *E. bonariensis* plants under salt stress, but only in those under the highest salt concentration in *E. sumatrensis*. The results obtained indicate that the two species are relatively tolerant to water deficit and medium salinity but are susceptible to high NaCl concentrations.

## 1. Introduction

Among environmental stressors, drought and salinity are the most detrimental to agricultural production, especially in arid and semi-arid areas. Water availability is the most important resource for plant growth and reproduction and the most limiting factor for agriculture in many areas of the world [1]. Secondly, salinisation affects almost 20% of irrigated land [2], and may extend to more than 50% of the world’s total irrigated areas by 2050 [3]. For this reason, an increasing number of studies are being conducted on responses to drought and salinity not only in model plants, but also in crops and their wild relatives [4,5,6,7,8]. Although unravelling functional responses to environmental factors in invasive plants is gaining interest [9], analysis of stress tolerance and mechanisms underlying stress responses are scarce in such species [10,11,12,13,14,15].

The two species under study belong to the genus *Erigeron* sect. *Conyza* and are invasive in the Mediterranean basin. As all members of this section, they have an American origin, but are world-wide invasive weeds [16]. *Erigeron bonariensis* L. (syn. *Conyza bonariensis* (L.) Cronquist) and *E. sumatrensis* Retz (syn. *Conyza sumatrensis* (Retz) E. Walker) together with *E. canadensis* L. are widespread in Europe [17] and this secondary distribution range is predicted to continuously increase under the present climatic conditions [18]. *E. bonariensis*, the hairy fleabane, is native to areas with a temperate climate in South America. It was first described in Argentina and reported in Europe dating back to 1700 and Australia at the middle of the 19th century [19]. Its European distribution is mainly Mediterranean, but it is expanding toward the north [20] and east of the continent [21]. It prefers undisturbed habitats on waste land, fields, along roadsides, in vineyards, and in orchards. When occurring as a weed, it is mostly problematic for perennial crops. As its seeds are very sensitive to soil burial [22] cultivation in annual crops buries most of the seeds produced and hinders emergence [20]. The species has pioneering traits, characteristic to the early successional stage, and is tolerant to infertile, saline, and sodic soils [20]. The second species under study, *E. sumatrensis*, with the common name tall or Sumatran fleabane, was recognized as a distinct species at the beginning of the 19th century [23] but was first reported in Europe only 1 century later [24]. It is now widespread in south-eastern Asia, Indonesia and the Philippines [19] and throughout western Europe and around the Mediterranean basin [20]. This species is usually associated with disturbed areas, being a successful colonizer taking advantages of disturbances [25]. It grows on open cultivated fields, roadsides, disturbed wetlands, and wastelands. In a comparative study with the horseweed (*E. canadensis*), the Sumatran fleabane was reported as an early to mid-successional species, posterior to colonization of the first [26]. It is considered as tolerant to infertile, saline, and sodic soils [20], but autumn frosts or spring droughts trigger a high seedling mortality [26].

*E. bonariensis* and *E. sumatrensis* are two of the most problematic, noxious, invasive and widespread weeds in different cropping systems around the world, especially in no-till farming systems, due to the persistence of the seeds in the soil seedbank and the lack of soil disturbance, which allows rosette plantlets to grow without being removed [27,28,29]. They are very competitive for resources, causing important yield losses in many crops, as soybean, cotton, corn, cereals, legumes, forages, fruit, and vegetable crops [27,28]. Both species have developed resistance to herbicides and complicate crop management [30,31]. *C. sumatrensis* has evolved resistance to herbicides from groups 2 (flazasulfuron, penoxulam), 4 (2,4-D), 9 (glyphosate), 14 (saflufenacil), and 22 (diquat, paraquat) and *C. bonariensis* to herbicides from groups 2 (chlorsulfuron), 5 (atrazine, simazine), 9 (glyphosate), and 22 (diquat, paraquat) [31].

Besides their negative impacts on crops and natural vegetation due to direct competition, the two species also play a role in transmission of pests and diseases [20]. In the last two decades several studies have been published on their biology and ecology [22,25,28,32], genetic variation [33], allelopathic effects [34], or resistance to pesticides [35,36,37,38]. Moreover, their seed germination ecology was analysed [22,29,39,40] but to our knowledge tolerance to abiotic stresses such as drought and salinity in different growth stages were not previously investigated.

The present study was conducted to evaluate the tolerance of *E. bonariensis* and *E. sumatrensis* to salt stress and water deficit during seed germination and vegetative growth and to examine the main mechanism of their stress responses. The objectives were: (i) to analyse the germination pattern of seeds of these species under salt stress at increasing concentrations of NaCl and osmotic stress induced by iso-osmotic concentrations of PEG; (ii) to evaluate their growth parameters under salt and severe water stress; (iii) to estimate the photosynthetic pigments degradation; (iv) to analyse the ionic homeostasis, and (v) to quantify oxidative stress markers and antioxidants.

## 2. Results

### 2.1. Effects of NaCl and PEG on Seed Germination

**Figure 1:**
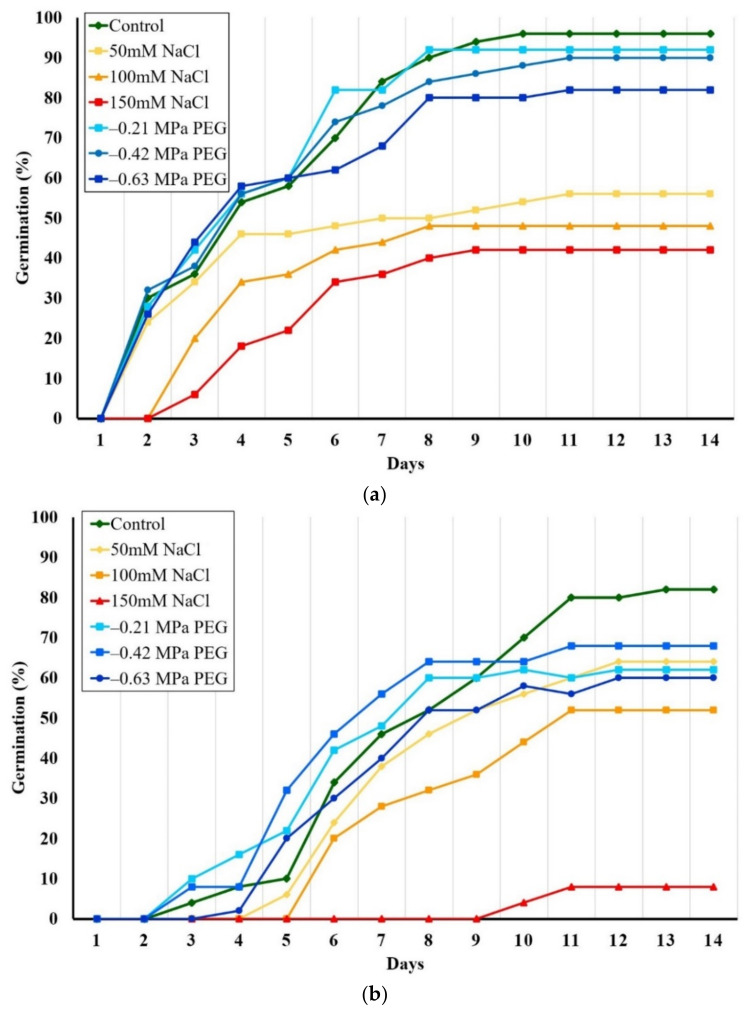
Evolution of seed germination as cumulative germination percentages over 14 days, in the presence of increasing isosmotic NaCl and PEG concentrations: (**a**) *Erigeron bonariensis*; (**b**) *E. sumatrensis*.

Seeds of the two species germinated within 2 weeks, a faster germination registered for *E. bonariensis* (Figure 1). The highest percentages of germination were found in the control, reaching 96% in *E. bonariensis* and 82% in *E. sumatrensis*. Salinity strongly affected both species. Under 50 mM salt concentration, only 56% of seeds of *E. bonariensis* and 64% of *E. sumatrensis* germinated. The reduction was stronger at higher NaCl concentration, reaching a minimum under 150 mM NaCl of 42% germination in the first species, but only 16% in the second. On the contrary, PEG had only a smaller effect on seed germination in the two species.

**Figure 2:**
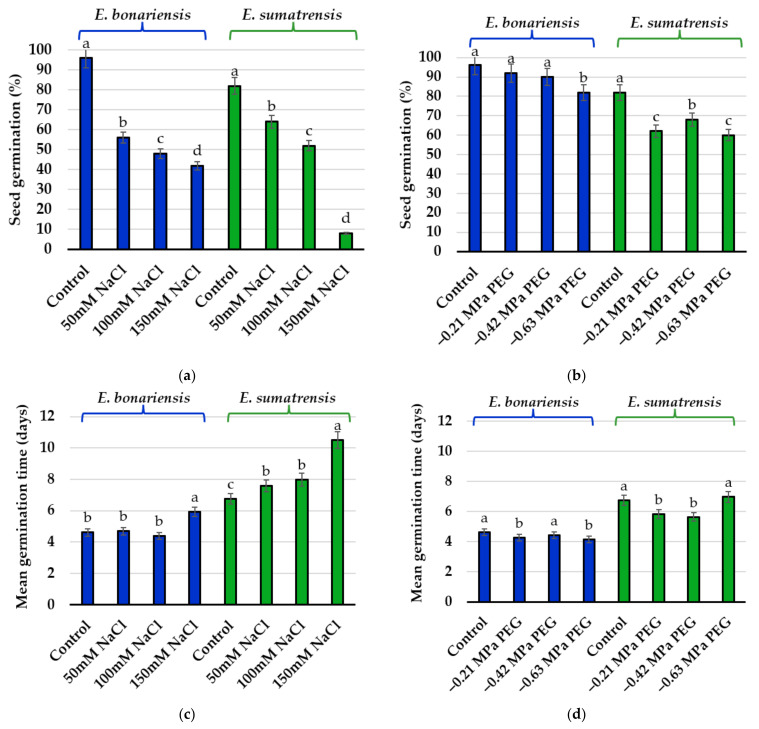
Final germination percentages and mean germination time after 14 days in the presence of increasing concentrations of NaCl and isosmotic concentrations of PEG: (**a**) germination percentage in *Erigeron bonariensis* and *E. sumatrensis* under NaCl stress; (**b**) germination percentage in *E. bonariensis* and *E. sumatrensis* under isosmotic concentrations of PEG; (**c**) mean germination time in *E. bonariensis* and *E. sumatrensis* under NaCl stress; (**d**) mean germination time in *E. bonariensis* and *E. sumatrensis* under isosmotic concentrations of PEG. Bars represent mean with SE, n = 5. Different lowercase letters indicate significant differences between treatments, within each species, according to the Tukey test (α < 0.05).

The one-way ANOVA performed on final germination percentages indicated a small but significant reduction only in the seeds of *E. bonariensis* from the treatment with the PEG concentration equivalent to 150 mM NaCl (−0.63 MPa). In *E. sumatrensis*, this reduction was significant under all PEG concentrations, but not as strong as for NaCl (Figure 2).

Of the three calculated germination indices, the largest oscillations among treatments were registered for germination indices (GIs) in both species (Table 1). GI values in *E. bonariensis* varied from wide limits, between 4.4 at a concentration of 150 mM NaCl and 69.0 in the control (without salt or water stress). In *E. sumatrensis*, the lowest value of GI was also noted at the concentration of 150 mM NaCl (0.4), and the highest in the control (31.9). The higher GI values registered in *E. bonariensis* indicate a greater rate of germination compared with *E. sumatrensis* in the absence of stress and both in the treatments with saline solutions and water stress. The values for speed of emergence index (SE) were more compact in *E. bonariensis* (between 14.3 and 42.9) and more dispersed in *E. sumatrensis* (between 3.3 and 50.0). The results of this index in *E. sumatrensis* were surprising especially due to the low value of the control (4.9), but high in salt concentration treatment of 150 mM NaCl (50.0) and even 100 mM NaCl (38.5). Coefficients of germination speed (CRG) registered higher values in *E. bonariensis* compared with *E. sumatrensis*. In both species, the lowest values were recorded at the salt concentration of 150 mM NaCl.

### 2.2. Effects of Salinity and Water Stress on Plant Growth

**Figure 3:**
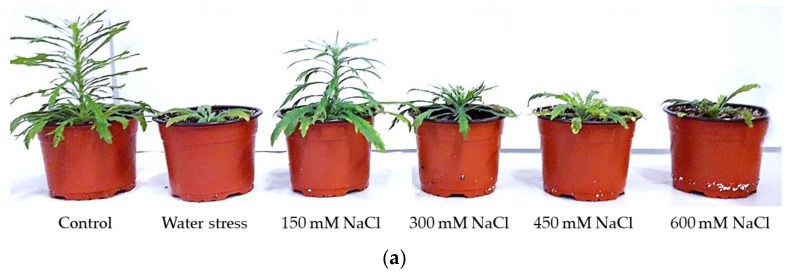

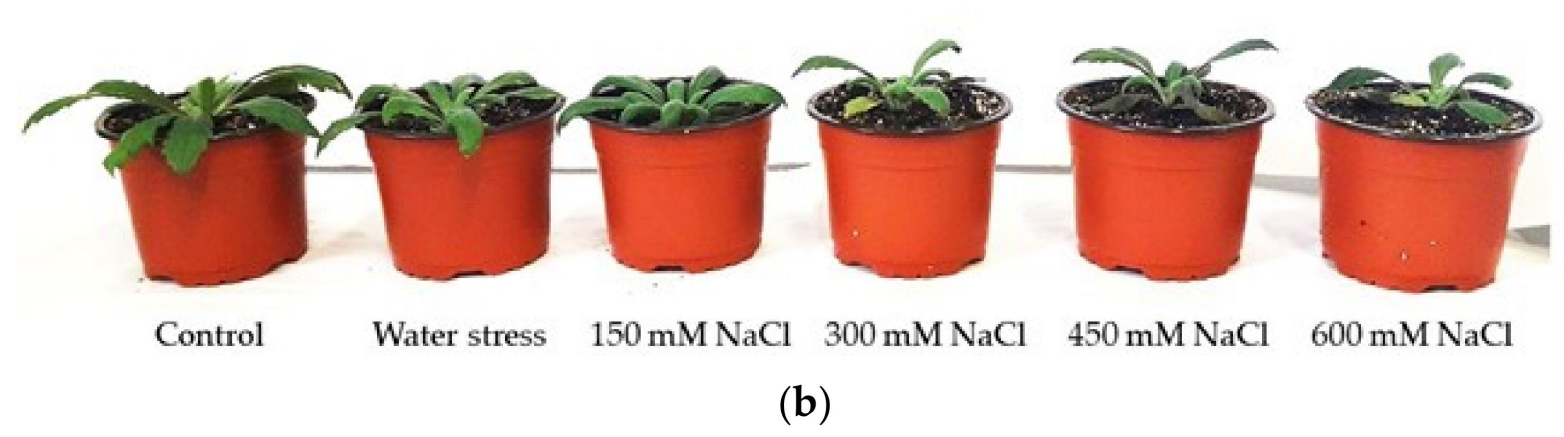
Images of plants after 1 month of treatments: (**a**) *Erigeron bonariensis*; (**b**) *E. sumatrensis*.

The stress treatments induced a reduction in vegetative growth in both species. The growth inhibition at the end of the treatments was more pronounced in *E. bonariensis*. However, in both species, the greatest reduction in growth occurred in the plants subjected to the highest salt concentration (Figure 3).

A two-way ANOVA was performed considering the factors species, treatment, and their interaction for all morphological traits analysed (Table 2). The strongest effect was that of treatment for all parameters except leaf area, where the data showed a large variability and the variation between treatments was not significant. The effect of species was also significant for all parameters, except leaf water content, but not as strong as that of treatments, as shown by the lower percentages of the sum of squares.

**Figure 4:**
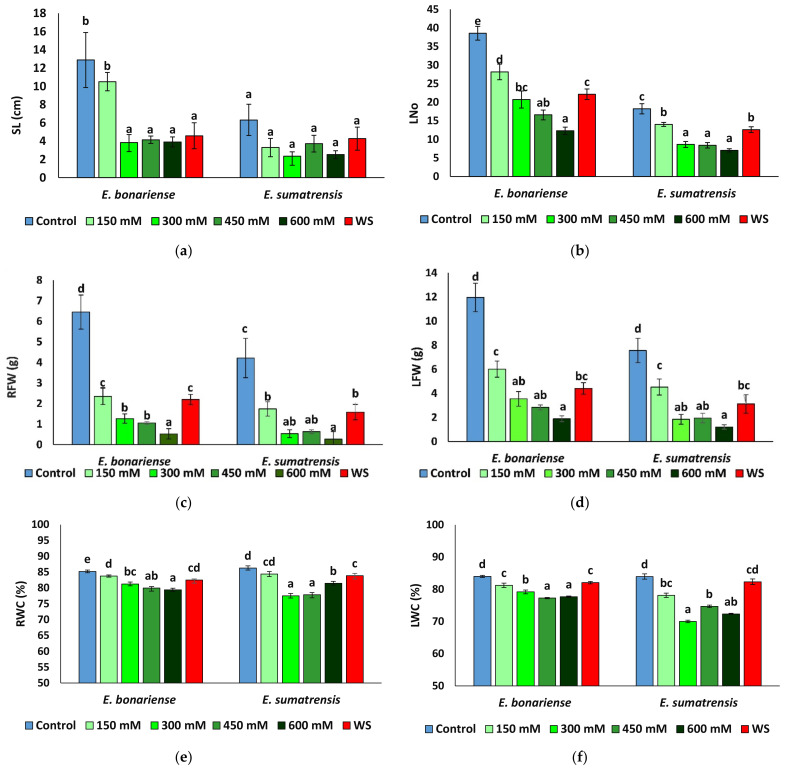
Growth parameters in the two *Erigeron* species after 1 month of water stress (WS) and treatments with NaCl at the indicated concentrations: (**a**) stem length (SL); (**b**) number of leaves (Lno); (**c**) root fresh weight (RFW); (**d**) leaf fresh weight (LFW); (**e**) root water content (RWC), (**f**) leaf water content (LWC). Means ± SE, n = 7. Same letters indicate homogeneous groups between treatments for each species, according to the Tukey test (α = 0.05).

Stem length was significantly reduced in *E. bonariensis* plants subjected to stress, with the exception of those at the NaCl concentration of 150 mM that did not vary significantly with respect to the control. In *E. sumatrensis* the reduction was not significant (Figure 4a). The mean leaf area showed large individual variability, but only small differences between plants of the different treatments, not significant in the two species (data not shown). In contrast, the leaf number decreased in the two species under stress, and the lowest leaf number was found in plants of the 600 mM NaCl treatment in the two species (Figure 4b). Under the stress treatments, plants of the two species showed a significant reduction in root (Figure 4c) and leaf (Figure 4d) fresh weight, and except for the lowest NaCl concentration applied, the effect of the saline treatments was stronger than that of the severe water stress. The greatest reduction was recorded in the 600 mM NaCl treatment, with a decrease in root fresh weight by more than 90% and of leaf fresh weight by more than 80% in both species. Saline treatments with concentrations higher than 300 mM NaCl had a stronger effect on root and leaf water content than water stress in the two species, but the variation in leaf water content in *E. sumatrensis* was not significant (Figure 4e,f).

### 2.3. Effect of Stress Treatments on Ion Homeostasis

Concentration of monovalent cations Na^+^ and K^+^ and that of the anion Cl^−^ was quantified at the end of the treatments in roots and leaves. The two-way ANOVA performed indicated a strong effect of the treatment, with exception of foliar K^+^. The effect of species was also significant, with exception of K^+^ in leaves and that of Cl^−^ in roots. The interaction of the two factors, species, and treatment was significant for K^+^ and Na^+^ in roots, indicating differences in the patterns of variations of these cations in the two species (Table 3).

**Figure 5:**
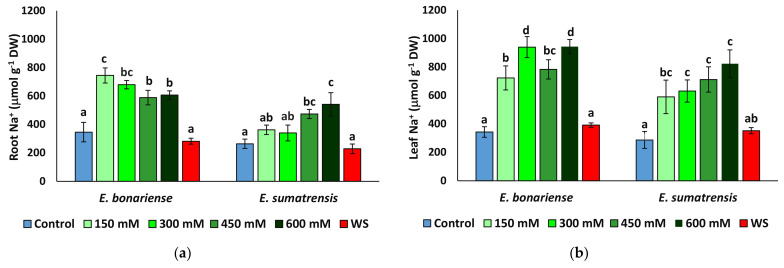

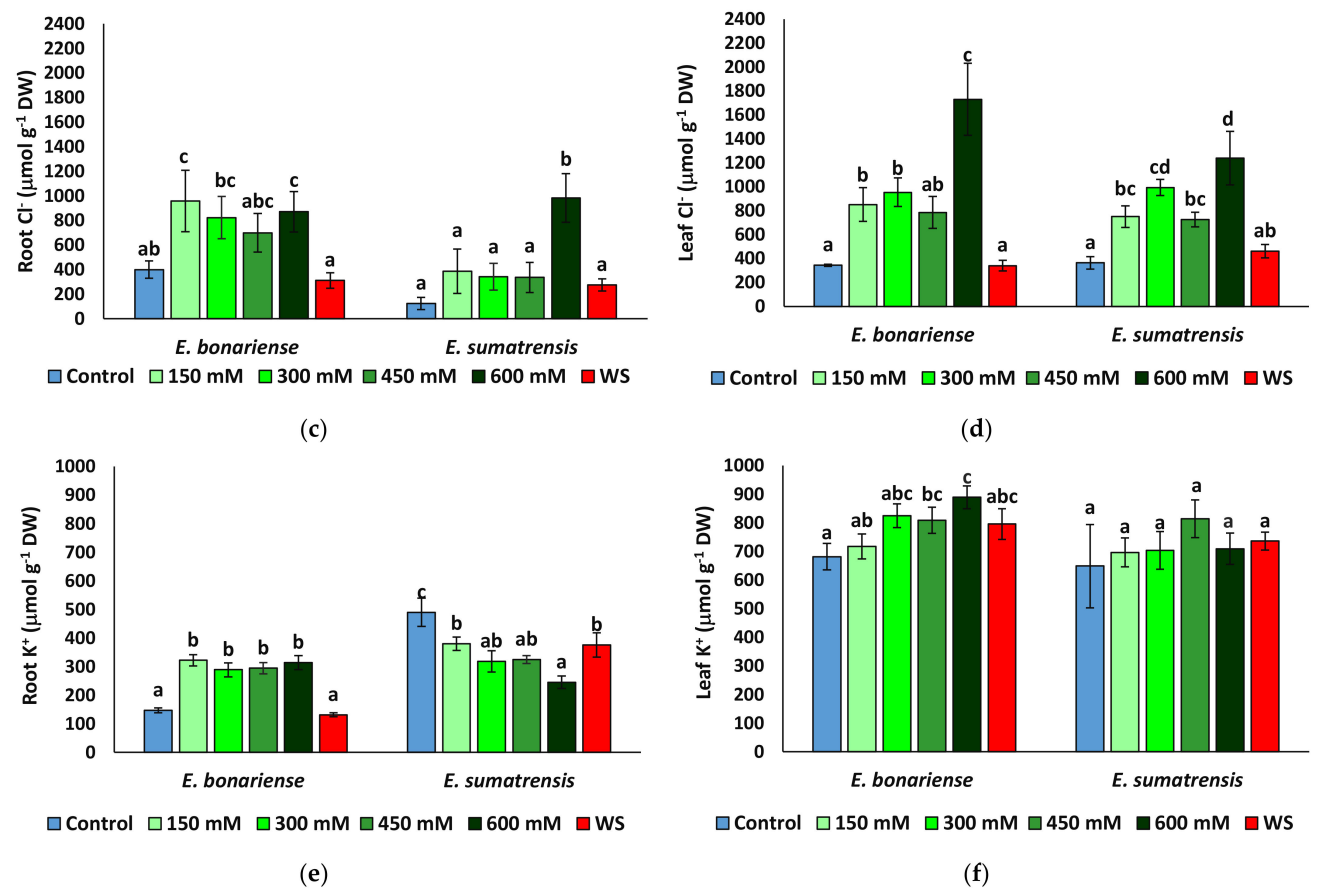
Ion contents in roots and leaves of *Erigeron bonariensis and E. sumatrensis* plants after 1 month of treatments with the indicated NaCl concentrations or 1 month of water stress (WS) (mean ± SE, n = 7): (**a**) Na^+^ in roots; (**b**) Na^+^ in leaves; (**c**) Cl^−^ in roots; (**d**) Cl^−^ in leaves; (**e**) K^+^ in roots; (**f**) K^+^ in leaves. Same letters indicate homogeneous groups between treatments for each species, according to the Tukey test (α < 0.05).

As expected, Na^+^ levels increased in plants subjected to saline treatments, but not in those of the water stress treatment (Figure 5a,b). Although the pattern of Na^+^ variation in leaves was similar in the two species, reaching maximum values under higher NaCl concentrations, it differed at the root level. In *E. bonariensis* plants accumulated the highest Na^+^ concentrations in the 150 mM NaCl treatment and its level decreased slightly in the roots of the 450 and 600 mM NaCl plants, while in *E. sumatrensis* the increase was gradual, with Na^+^ concentrations being significantly different from the control only in the plants of the 450 and 600 mM NaCl treatments. A similar trend was observed for Cl^−^ in roots, with the highest concentrations in *E. bonariensis* in the 150 mM NaCl plants, while in *C. sumatrensis* a significant difference from the control was recorded only in plants subjected to the highest salt concentration (Figure 5c). The lowest Cl^−^ values in roots and leaves were observed in both species in the control and water stress treatment plants. In the two species in leaves, Cl^−^ increased significantly in all salt treatments, with a peak in plants treated with the 600 mM NaCl solution (Figure 5d). Interestingly, in roots of *E. bonariensis* K^+^ levels increased slightly in plants of the saline treatments and remained unchanged with respect to the control in water-stressed plants. In contrast, K^+^ levels in *E. sumatrensis* roots decreased in all stress treatments, with the lowest values detected in plants from the 600 mM NaCl treatment (Figure 5e). K^+^ levels in leaves of *E. bonariensis* increased while those of *E. sumatrensis* did not change (Figure 5f).

### 2.4. Effect of Stress on Biochemical Parameters

Chlorophyll a and b, proline, malondialdehyde (MDA), total phenolic compounds, and total flavonoids were quantified in fresh leaf material after 1 month of stress treatments. A two-way ANOVA indicated that only some of these parameters showed significant variation according to species or treatment (Table 4). Proline variation showed significant variation according to treatment and species and their interaction. The variation in total phenolic compounds was mainly related to the effect of species. Photosynthetic pigments, total flavonoids, and MDA had very high residual values, and their variation did not depend on either factor.

**Figure 6:**
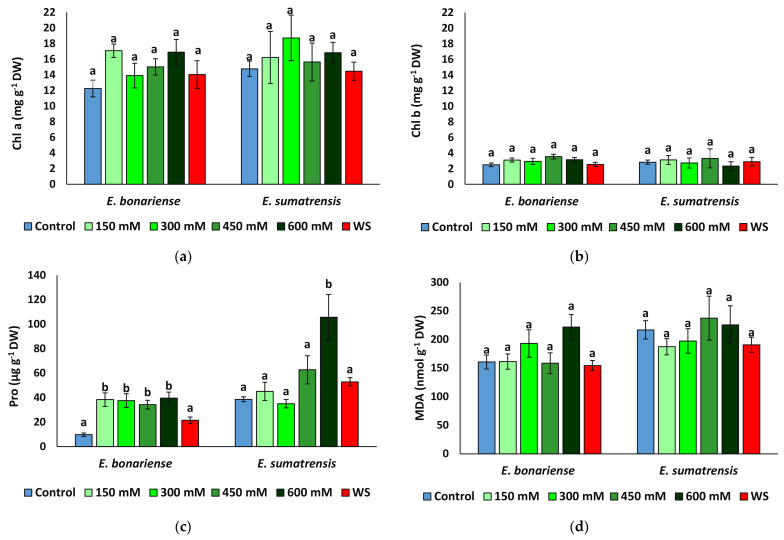

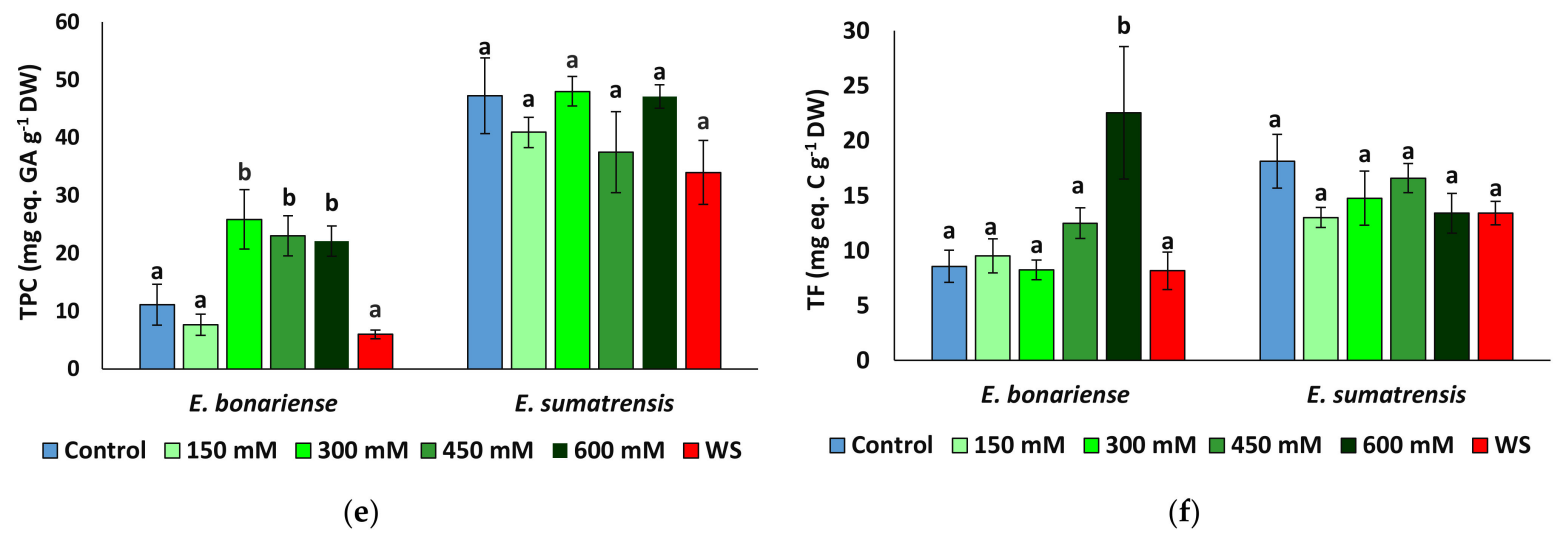
Concentrations of biochemical compounds analysed in the leaves of *Erigeron bonariensis and E. sumatrensis* plants after 1 month of treatment with the indicated NaCl concentrations or 1 month of water stress (WS) (mean ± SE, n = 7): (**a**) chlorophyll a (Chl a); (**b**) chlorophyll b (Chl b); (**c**) proline (Pro); (**d**) malondialdehyde (MDA); (**e**) total phenolic compounds (TFCs); (**f**) total flavonoids (TFs). Same letters indicate homogeneous groups between treatments for each species, according to the Tukey test (α < 0.05).

Chlorophylls showed only small fluctuations unrelated to treatment, and concentrations were similar in leaves of plants of the two species (Figure 6a,b). Proline concentration quadrupled to 39 µmol g^−1^ DW (dry weight) in *E. bonariensis* plants subjected to salt stress, but not in those subjected to water stress, which did not vary significantly from those in the control. In *E. sumatrensis*, proline increased significantly only in plants of the 600 mM NaCl treatment, reaching 105 µmol g^−1^ DW (Figure 6c). However, this represents only a 2.7-fold increase, as the control plants have high proline levels of 38 µmol g^−1^ DW. MDA (malondialdehyde) concentrations did not largely vary between treatments (Figure 6d), and significant variation was found only for total phenolic compounds in salt-stressed *E. bonariensis* plants and total flavonoids in plants of the same species from the 600 mM NaCl treatment (Figure 6e,f).

### 2.5. Regression and Correlation Analyses

**Figure 7:**
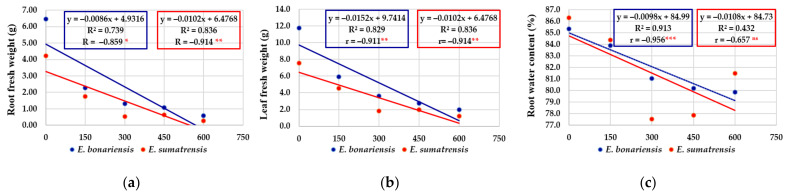

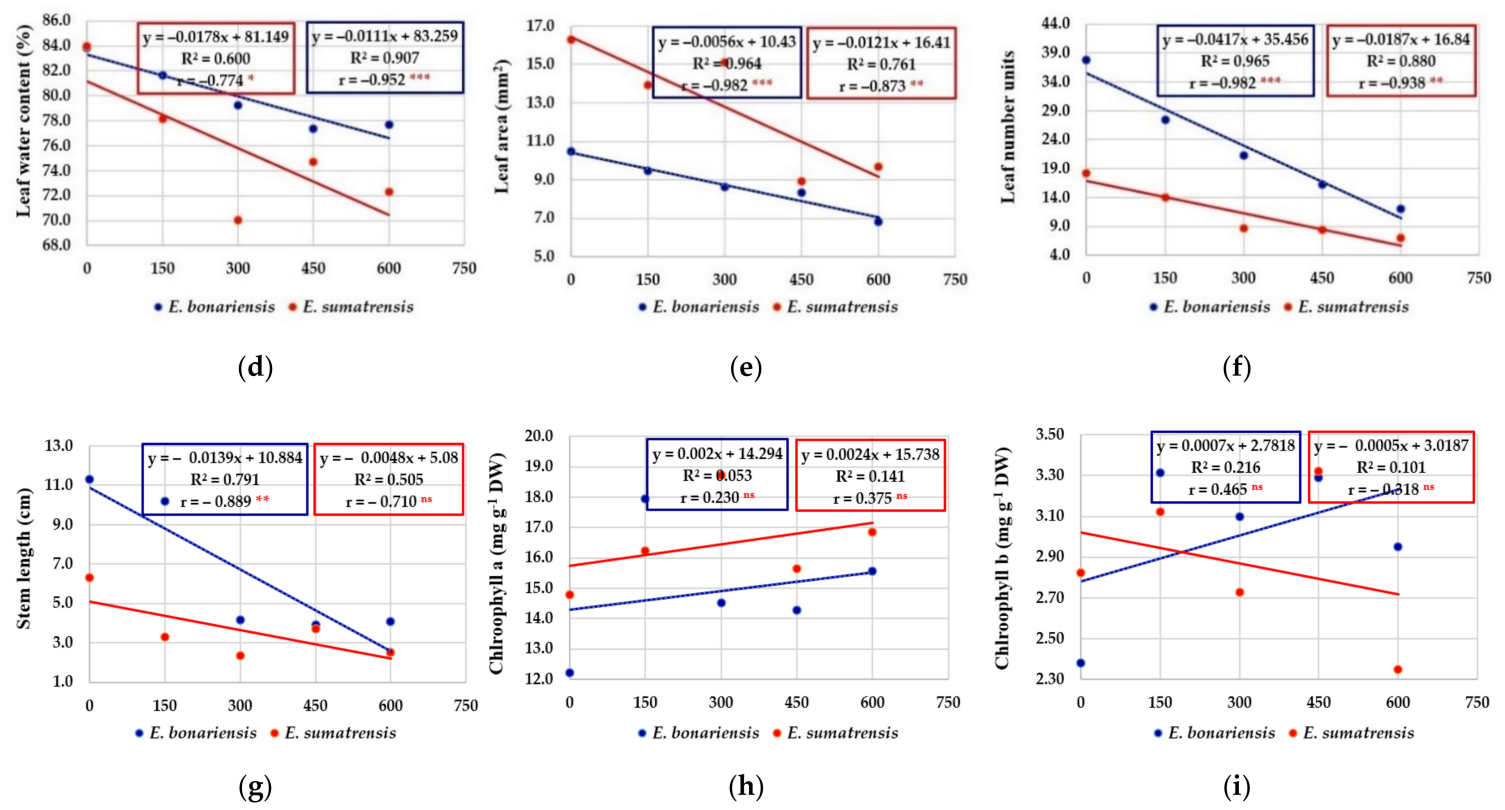
The effect of salt concentration (NaCl solution at 0, 150, 300, 450, and 600 mM) on the morphological characteristics and concentrations of chlorophyll analysed in *E. bonariensis* and *E. sumatrensis* species: (**a**) Root fresh weight; (**b**) Leaf fresh weight; (**c**) Root water content; (**d**) Leaf water content; (**e**) Leaf area; (**f**) Leaf number units; (**g**) Stem length; (**h**) Chlorophyll a; (**i**) Chlorophyll b. In each figure are presented regression equation, the coefficient of determination (R^2^), and the coefficient of correlation (r). For r, ‘ns’ superscript means not significant, and the asterisks indicate the degree of significance: * <0.05, ** <0.01; *** <0.001.

For the morphological characteristics of the plants, the regression equations had negative values, and the regression line had an obvious descending tendency (Figure 7a–g). As expected, the increase in salt concentrations had a negative effect on the elements that contribute to plant biomass. The coefficients of determination (‘R^2^’) illustrated a consistent contribution of the independent variable, considering the solution of NaCl (at 0, 150, 300, 450, and 600 mM concentrations), salinity treatments as a stress factor, on the dependent variable represented by stem length, number of leaves, leaf area, fresh and dry weight of roots and leaves, but also the percentage of water content in roots and leaves. Thus, the information provided by simple linear regressions is suggestively supplemented by R^2^ values as the proportion of variance in the dependent variable that is predicted or explained (accounted) by the statistical model. High values of R^2^ in characters such as number of leaves, leaf area, fresh leaf weight, etc. were recorded. For instance, for the number of leaves per plant in *E. bonariensis*, 96.5% of the variance is predicted by the salt concentration and only the very small difference of 3.5% is unexplained by the model. *E. sumatrensis* also increased, saline stress has a large effect on the morphological trait represented by the number of leaves per plant (88.0% of the total variance). Finally, the correlation coefficients (‘r’) provide clear information on how saline stress is negatively correlated with the morphological characters analysed. Practically all their values are significant at different levels of significance, with two exceptions, both recorded in *E. sumatrensis*, for the percentage of water content in roots and for stem length (where r values were symbolized by ‘ns’, i.e., non-significant).

**Figure 8:**
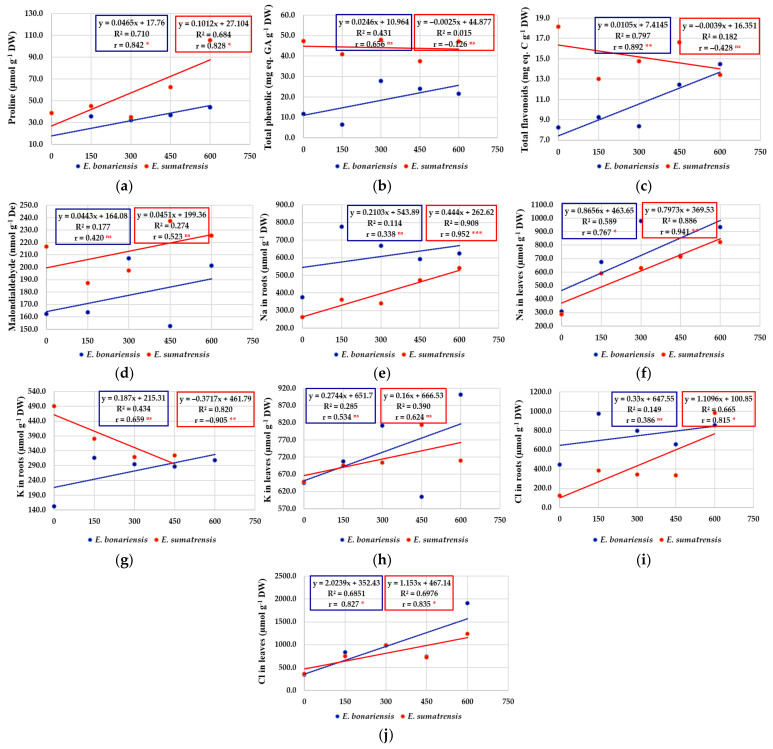
The effect of salt concentration (NaCl solution at 0, 150, 300, 450, and 600 mM) on the concentrations of proline, total phenolics, and flavonoids and monovalent ions measured in *E. bonariensis* and *E. sumatrensis* species: (**a**) Proline; (**b**) Total phenolic compounds; (**c**) Total flavonoids; (**d**) Malondialdehyde; (**e**) Na in roots; (**f**) Na in leaves; (**g**) K in roots; (**h**) K in leaves; (**i**) Chlorine in roots; (**j**) Chlorine in leaves. In each figure are presented regression equation, the coefficient of determination (R^2^), and the coefficient of correlation (r). For r, ‘ns’ superscript means not significant, and the asterisks indicate the degree of significance: * <0.05, ** <0.01; *** <0.001.

In contrast to the morphological characteristics of plants, negatively correlated with salt concentrations, biochemical parameters analysed in plants (chlorophyll content, monovalent ions, proline, malondialdehyde, total phenolic compounds, and total flavonoids) were almost entirely positively correlated. As a result, in general, for these characteristics the regression equation was positive and the regression line had an ascending allure (Figure 8). The coefficients of determination and correlation provide useful information on the response of plants to saline stress and how plants concentrate certain chemical compounds in response to salinity levels. In most cases, the correlation coefficients are significant. Exceptions (r values non-significant) include the content of chlorophyll a and b, total phenolic, malondialdehyde, K^+^ in leaves, etc. There were also some different situations when for certain characteristics the two species reacted differently. These are highlighted more suggestively by the regression line, which in *E. bonariensis* has an ascending allure, and in *E. sumatrensis* a descending one, to the following characteristics: chlorophyll b (Figure 7i); total flavonoids (Figure 8c); and K^+^ in roots (Figure 8g).

**Figure 9:**
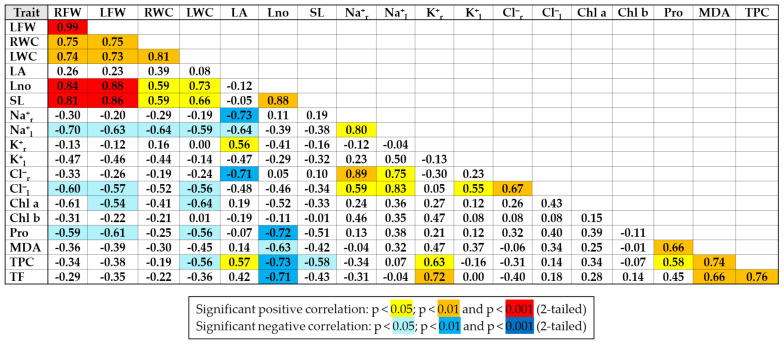
Pearson correlations between the main characteristics analysed in *E. bonariensis* and *E. sumatrensis*, on the ensemble of the experiment. Abbreviations: RFW—fresh weight of roots; LFW—fresh weight of leaves; RWC—water content of roots; LWC—water content of leaves; LA—leaf area; Lno—number of leaves; SL—stem length; Na^+^_r_—sodium in roots; Na^+^_l_—sodium in leaves; K^+^_r_—potassium in roots; K^+^_l_—potassium in leaves; Cl^−^_r_—chlorine in roots; Cl^−^_l_—chlorine in leaves; Chl a—chlorophyll a; Chl b—chlorophyll b; Pro—proline; MDA—malondialdehyde; TPC—total phenolic compounds; TF—total flavonoids.

Pearson correlations were performed on all data of the two species (Figure 9). Significant positive correlations were recorded between growth parameters except the leaf area. As expected, the strongest positive correlations were found for root and leaf fresh weight and root and leaf water content. Strong positive correlations were also found between Na^+^ and Cl^−^ concentrations in roots and leaves and between the concentrations of these two ions. Total phenolic compounds (TPCs) were strongly correlated with total flavonoids (TFs) and MDA. Significant negative correlations were established between Na^+^ and Cl^−^ in leaves and growth parameters, especially root fresh weight. Plant leaf number was strongly negatively correlated with proline (Pro), total phenolics (TFCs) and flavonoids (TFs) and to a lesser extent with MDA. Chlorophyll variation was not correlated with that of other parameters analysed.

### 2.6. Multivariate Analysis of Data

**Figure 10:**
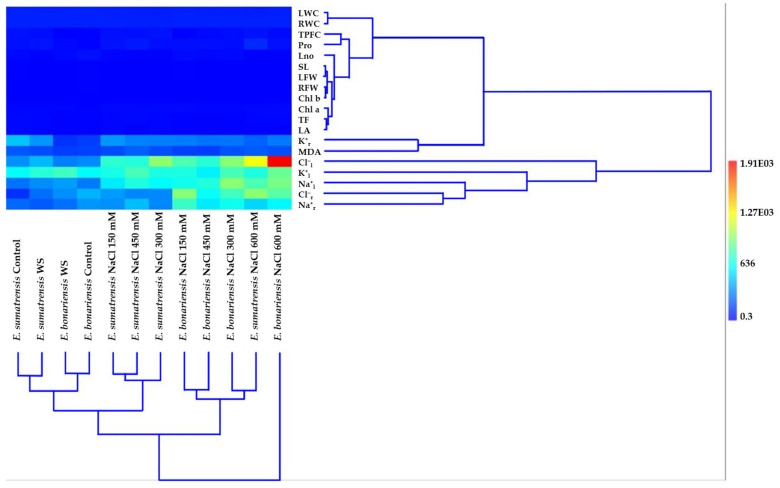
Multivariate analyses for the studied characteristics: Hierarchical clustering, algorithm Ward’s method, similarity index Euclidean of the two species of *Erigeron (E. bonariensis* and *E. sumatrensis*). Abbreviations as in Figure 9.

Multivariate analysis (hierarchical clustering using Ward’s method, Euclidean similarity index) performed with mean values of all parameters highlights interesting relationships both for the interaction of *E. bonariensis* and *E. sumatrensis* with the applied stress treatments (column dendrogram) and for the approach or distance of some of the analysed characteristics (row dendrogram), their heatmap (Figure 10).

Related to the two largest clusters in the column dendrogram of species, the one on the right is represented only by *E. bonariensis* at the highest salt concentration (600 mM NaCl) away from the other combinations of species × treatment, while the other cluster includes all combinations, grouped into two subclusters. Of the two subclusters, the one on the right has two subclusters, each with two branches: one containing *E. bonariensis* at 150 and 450 mM NaCl, and the other *E. bonariensis* at 300mM and *E. sumatrensis* at 600 mM salt. The left subcluster has two other subclusters, with subdivisions that ensure a harmonious grouping of species and treatments. Thus, there is close proximity in a small subcluster between the control and water stress in *E. sumatrensis*, and in the other small subcluster between the control and water stress in *E. bonariensis*. The three combinations of *E. sumatrensis* with NaCl concentrations of 150 mM, 450 mM, and 300 mM, respectively, are also grouped in a quite homogeneous subcluster.

In the row dendrogram of the analysed characteristics, there are several small subclusters consisting of two or three characteristics (i.e., SL and LFW; RFW and Chl b; TF and LA, both with Chl a), which together form a distinct group or subcluster, and is very compact. This subcluster has a single pruning line above, and four nodes corresponding to the ramification positions under the pruning line. A very close relationship also appears between LWC and RWC, this was also previously confirmed by the significant correlation coefficient between the two characteristics. Of the two large clusters in the upper one, a distinct subcluster of those mentioned above is represented by the branch on which K^+^_r_ and MDA are positioned. In the lower cluster, the closest connection is recorded between the pair Cl^−^_r_ and Na^+^_r_, then these with K^+^_l_, and above with Cl^−^_l_.

Both dendrograms highlight clusters and their corresponding cells in the heat map, emphasizing especially the hot cell (red) at the intersection of the vertical alignment represented by *E. bonariensis* at the highest salt concentration (600 mM) and the horizontal alignment represented by Cl^−^.

## 3. Discussion

Successful germination is a key phase in the biological cycle of plants, and it is critical for plant propagation and adaptation to environment [41]. Therefore, germination has direct effects on the fitness of populations, ecological niches occupied, the range of distribution, and the evolutionary potential of species [12,42]. The success of biological invasions depends largely on their reproductive traits, especially seed production and germination in plants [42,43,44,45]. Previous studies have often correlated seed germination traits with the invasiveness of plants; for example, invasive alien species have been found to produce a higher number of tiny seeds [46,47], with a high germination rate [48,49,50]. Moreover, their germination is more successful in more varied environmental conditions than that of native, non-invasive congeneric species [48,51]. In the case of weeds, knowledge of the effects of environmental factors on seed germination and seedling emergence is important not only for understanding species biology, but also for risk assessments and for establishing management strategies in the context of global changes [12]. Thus, knowledge of the ecology and germination of invasive plant species is essential for the development of effective weed management systems both economically and environmentally. Information on the ecology of invasive weed species, their dynamics, rates of adaptation and response to environmental factors is needed to prevent potential new invasions and mitigate the long-term impacts of invasive weed species [52,53,54].

The two *Erigeron* species analysed here follow the “ruderal strategy” of producing a large number of small seeds, which can germinate quickly [42]. In addition, the flowering period lasts several months and the sequentially developing flowers are self-compatible and self-pollinating [26,55]. The number of seeds reported in *E. bonariensis* was up to 375,500 seeds per plant [56], and the seeds of both species are light and attached to a pappus with anemochore dispersal over large distances [57]. Other traits of these species, such as low seed dormancy, a high emergence rate, and the evolution of increasing resistance to glyphosate, make them particularly serious invasive weed [22,29,40].

Optimal germination was found in the absence of stress: a higher germination percentage with a mean of 96% was found in the control treatment in *E. bonariensis*, while in *E. sumatrensis* only 82%, which are similar to those reported for the two species under optimal conditions [29,39]. However, slightly higher germination in the former species cannot be associated with higher invasiveness, as germination percentage is not considered a consistent predictor of invasiveness, more relevant is the speed of germination [42]. Invasive species are characterised by a short time to germination and rapid germination. In the two species analysed here, the germination speed was clearly higher in *E. bonariensis*, with a mean germination time (MGT) of 4.62, while in the other species the MGT was 6.76. Early and rapid germination ensures a mitigation or avoidance of competition, favouring the occupation of vacant germination niches [42].

Even in halophytes, or salt-tolerant plants, the optimal germination occurs mostly in the absence of stress [58] and in natural environments it usually occurs after periods of heavy rainfall, when soil salinity is alleviated [59]. Maximum salt tolerance for seed germination has been reported to vary between 1.7 and 0.26 M NaCl depending on the species and the environmental conditions [60].

Exotic species are considered to have greater germination plasticity [48]. Both *Erigeron* species maintained their germination under salt stress, and 42% of seeds of *E. bonariensis* germinated under the highest NaCl concentration tested of 150 mM NaCl. Higher salt concentrations were not tested in this study, but some reports indicated that seeds of species germinate up to 200 mM NaCl [61], being more tolerant to salinity at this stage than the closely related *C. canadensis* [62]. Germination in *E. sumatrensis* was also more affected in our experimental conditions by the highest salt solution applied, as only 16% of seeds germinated at 150 mM NaCl. Mahajan et al. [40] reported that seeds of this species did not germinate at 180 mM NaCl, although in a study comparing germination of seeds originating from different climatic conditions and different soil types, notable differences were found between populations [29]. Germination percentages such as those found in the presence of NaCl, although low considering the high number of seeds produced, can lead to new populations established under stress conditions, as has been reported in other species. For example, in *Iris pseudacorus* which is invasive in California, it has been reported that even such rare events can contribute to the spread of the species in highly saline environments [63]. The importance of phenotypic plasticity in germination traits as a determinant of the success of invasive species over native species was also reported by Paudel and Battaglia [64]. These authors evaluated the effects of elevated salinity on initial recruitment of the invasive woody species *Triadica sebifera* and two native woody species.

The standard method to test the osmotic effect on germination is by using different concentrations of polyethylene glycol (PEG), which mimics environmental drought conditions [65,66,67,68]. Germination of the two species proved to be tolerant to osmotic stress, no significant differences were obtained with respect to the control up to the highest PEG concentration in *E. bonariensis* where 92% of the seeds and in *E. sumatrensis* 60% of the seeds germinated, a considerably lower reduction than under NaCl. Germination time (MGT) did not vary from the control. Salt and osmotic stress tolerance were reported in other invasive weeds of different genera, such as, *Araujia* [15], *Amaranhus* [14], *Chenopodium* [69], and *Cenchrus* [70], which thrive in disturbed habitats (either altered by natural or anthropogenic forces) due to increased plant fitness through functional and adaptive traits [71]. The stronger effects of NaCl than of PEG can be explained by the ionic toxicity, as salinity has a toxic component in addition to the increased external osmotic potential that reduces water uptake during imbibition also produced by PEG. Excess sodium and chloride ions have toxic effects on embryo viability, induce alteration of the structure of enzymes and other macromolecules, damage cell organelles and the plasma membrane, and disrupt respiration, photosynthesis, and protein synthesis [60].

Invasive plants have usually a fast growth [72]. *E. sumatrensis* reaches a size over 2 m, as its common name tall fleabane indicates; whereas *E. bonariensis* is rarely more than 60 cm tall [20]. However, at the end of the treatments, the control plants of *E. sumatrensis* were shorter than that of the other species, indicating a slower rate of growth in the absence of stress. Growth was affected by water and salt stresses in the two species in a similar manner, both showing a marked reduction mostly in the salt treatments starting with the concentration of 300 mM NaCl. Although plants from the 150 mM NaCl and water stress treatments had a smaller size than those in the controls, with the exception of root fresh weight in *E. bonariensis*, the reduction did not surpass 50% in any of the parameters analysed, indicating a relative tolerance to mild salinity and drought in the two species.

One of the main mechanisms involved in the salt tolerance of plants is the control of ion transport by the uptake of toxic ions, Na^+^ and Cl^−^, and their compartmentalization in the vacuole, present mostly in dicotyledonous halophytes, or by reduction their absorption by roots and uptake enhancement, and accumulation of K^+^ in glycophytes and monocotyledonous halophytes [73,74]. K^+^ is an essential nutrient, playing a key role in many cellular and physiological processes in plants [75] but when Na^+^ is in excess a drop in its level occurs in salt susceptible plants. This is due to the competition between the two cations for the same transporters and binding sites, which causes an increase in the Na^+^/K^+^ ratio at levels that exceed the K^+^/Na^+^ selectivity of many K^+^ channels [76,77].

In our experimental conditions, as expected, Na^+^ and Cl^−^ concentrations increased in salt-treated plants of the two species. The K^+^ uptake was enhanced under salinity only in *E. bonariensis*, as shown by its higher levels in roots and leaves of salt-treated plants. On the contrary, in *E. sumatrensis* a small reduction in the levels of root K^+^ was found in the plants subjected to the salt treatments. However, levels of K^+^ were much higher in foliar tissue than in roots in this species in all treatments, indicating an active transport of K^+^ from roots to leaves. Ion levels did not vary in plants subjected to water stress, except for a smaller concentration of K^+^ in roots of water stressed plants of tall fleabane, but foliar K^+^ were stable.

Chlorophyll concentration usually decreases in the presence of high NaCl or severe drought in stress susceptible plants due to the inhibition of its biosynthesis and/or activation of chlorophyllase which degrades the photosynthetic pigments [78,79,80]. The lack of significant differences between the concentrations of photosynthetic pigments in plants from the different treatments indicate that the two species here analysed are relatively more tolerant than many other glycophytes which suffer a reduction in chlorophylls under stress [81,82,83].

A general mechanism of ensuring the osmotic balance under stress conditions is the accumulation of compatible solutes, or osmolytes. They are very diverse from a chemical point of view and are accumulated under a wide range of environmental stresses, having a role not only in osmoregulation, but also functioning as low-molecular weight chaperones, reactive oxygen species (ROS) scavengers or signalling molecules [84,85]. Proline is one of the most common compatible solutes [86], with additional antioxidant functions as in ROS scavenging, stabilising mitochondrial respiration enzymes, and defences against pathogens [86,87]. The pattern of proline accumulation was different in the two species here analysed; in *E. bonariensis* a 4-fold increase was registered in all salt treatments, in *E. sumatrensis* only plants subjected to the highest concentration of NaCl showed a significant increase of 2.7-fold in respect to the control. On the other hand, proline concentration in this latter species was considerably higher than in the other one in all treatments, values registered in the control plants were similar to those found in *E. bonariensis* in salt-stressed plants. Such high levels of proline in the absence of stress in *E. sumatrensis* may represent a constitutive mechanism of tolerance to stress. Under water stress proline concentration did not differ significantly from the control in either species indicating that the two species tolerate better drought than salinity, in agreement with their range of distribution mostly in dry climates but generally not on saline soils.

Abiotic stress is associated with increased production of reactive oxygen species (ROS), which when in excess may damage nucleic acids, lipids, and proteins and induce severe dysfunctions and even cell death [88]. Malondialdehyde (MDA) is a marker of membrane lipid peroxidation used to estimate the level of oxidative stress experienced by plants and to evaluate the plants’ susceptibility to different types of stress [89,90]. MDA concentrations showed only small fluctuations in stressed plants with respect to those from the control indicating that oxidative stress is not considerable in our experimental conditions. However, concentrations of total phenolics increased in plants of *E. bonariensis* treated with salt solution starting with 300 mM NaCl, whereas in *E. sumatrensis* only the concentration of antioxidant flavonoids increased at 600 mM NaCl. Phenolic compounds and especially flavonoids are strong antioxidants [88], the latter regarded as a secondary ROS scavenging system in plants suffering damage to the photosynthetic apparatus due to excess excitation energy [91].

## 4. Materials and Methods

### 4.1. Seed Collection

Seeds of *Erigeron bonariensis* were collected from horticultural crop fields that were not cultivated at the moment of seed collection, and seeds of *Erigeron sumatrensis* were collected from persimmon tree fields located in L’Alcúdia (L’Alcúdia, Valencia province, Spain) not treated with herbicides, in July 2021. *E. bonariensis* was the predominant weed species in the field in which it was collected, with 50% of coverage; while *E. sumatrensis* was found combined with other weed species, many of them nitrophilous, such as *Chenopodium album*, *Amaranthus retroflexus*, and *Portulaca oleracea*, representing *E. sumatrensis* 20% of weed coverage. Both species, *E. bonariensis* and *E. sumatrensis* are considered invasive in Spain.

### 4.2. Seed Germination

Germination of seeds was conducted in a growth chamber (model EGH1501HR from Equitec, Madrid, Spain) at 25 °C under an 11 h photoperiod. For each treatment, 100 seeds were sown in 5 standard Petri dishes of 9 cm diameter (20 seeds per plate), with filter paper moistened with 5 mL of distilled water for the control, aq, solutions of 50, 100, and 150 mM NaCl for testing the effect of salinity on germination and with iso-osmotic solutions of polyethylene glycol 6000 (PEG 6000). The proper concentration of PEG was calculated applying the Van’t Hoff equation [92]. Plates were sealed with parafilm to avoid evaporation and were incubated for 2 weeks, counting daily the number of germinated seeds, upon radicle emergence. Besides germination percentage (GP), the following indices of germination were calculated:

Germination index, GI [93]:

$$\mathrm{GI}=\frac{Number\;of\;germinated\;seeds}{Days\;from\;the\;first\;control}+\ldots +\frac{Number\;of\;germinated\;seeds}{Days\;from\;the\;last\;control\;}$$

Speed of emergence, SE (using Germination speed/Germinative energy) [94]:

$$\mathrm{SE}=\frac{Number\;of\;germination\;seeds\;in\;the\;first\;day\;of\;germination}{Number\;of\;germinated\;seeds\;in\;the\;last\;day\;of\;germination}\times 100$$

Coefficient of germination speed, CRG [95]:

$$\mathrm{CRG}=\frac{n1+n2+\ldots +nn}{\begin{array}{c} ++n+1+x+T+1+++++n+2+x+T+2+++++\\ \;an3xT3+\ldots +nnxTn\;\; \end{array}}\times 100$$

where:

n_1_ = number of seeds germinated in day 1 (T_1_); n_2_ = number of seeds germinated in day 2 (T_2_); n_n_ = number of seeds germinated in day n (T_n_).

### 4.3. Plant Growth and Stress Treatments

The plants used in the growth assays originated from the seeds germinated in the control treatment of the previous experiment. Seedlings were transferred from Petri dishes with filter paper moistened with 5 mL of water to 0.5 L pots (11 cm diameter and 10 cm depth) containing a substrate mixture of peat, perlite, and vermiculite (2:1:1), placed in plastic trays and watered twice a week with half-strength Hoagland solution [88]. The trays with the pots were maintained in a phytotron under long-day photoperiod conditions (16 h of light and 8 h of darkness), and temperatures of 23 °C during the day and 17 °C at night. Relative humidity ranged from 50 to 80%. After 1 month, the stress treatments were initiated. Pots containing individual plants were placed in different trays for each treatment and species (7 pots/tray). Irrigation was administered twice a week, those in the control treatment with half-strength Hoagland nutrient solution added to the trays (1.5 L per tray), and plants in the salt stress treatments with the same volume of nutrient solution contained NaCl in final concentrations of 150, 300, 450, and 600 mM. The treatments were finalised after 1 month, when the plant material was sampled, quantifying the following growth parameters: increase in stem length, number of leaves, leaf area (measured with Digimizer software – Digimizer v.5.4.7 ® (MedCalc Software Ltd., Ostend, Belgium, 2020)), fresh and dry weight, and percent of water content, calculated as [96]:

WC% = [(FW−DW)/FW] × 100

where: WC%—water content percentage; FW—leaf fresh weight; DW—dry weight.

### 4.4. Ion Quantification

The monovalent ion content was determined at the end of the assay in the roots and leaves of all sampled plants, following the Weimberg extraction protocol [85], which consists of adding 25 mL of water to 0.15 g of dried and ground material and, after homogenization, incubation of the samples for 1 hour at 95 °C in a water bath. The samples were then filtered through filter paper (particle retention 8–12 µm), and the sodium and potassium contents were quantified using a PFP7 flame photometer (Jenway Inc., Staffordshire, UK). Chlorides were measured with a chloride analyser (Sherwood, model 926, Cambridge, UK).

### 4.5. Photosynthetic Pigments

The concentration of chlorophyll a (Chl a) and chlorophyll b (Chl b) was estimated according to the classical protocol of Lichtenthaler and Wellburn [97] using 0.05–0.10 g of fresh leaves. In the presence of liquid nitrogen, 1 mL of ice-cold 80% acetone was added to each sample, which was shaken overnight in the dark at 4 °C. After a 10 min centrifugation at 13,300 g and 4 °C, the supernatants were separated and absorbance was measured at 470, 646, and 663 nm. The following equations were used to calculate pigment concentrations [97]:

Chl a (µg/mL) = 12.21 × (A663) − 2.81 × (A646)

Chl b (µg/mL) = 20.13 × (A646) − 5.03 × (A663)

Final concentrations were expressed in mg·g^−1^ DW.

### 4.6. Proline Quantification

Proline (Pro) was quantified according to the method of Bates et al. [98] from 0.05 to 0.10 g of fresh leaf material extracted in 3% aqueous sulfosalicylic acid. The samples were then mixed with acid ninhydrin solution and incubated at 95 °C for 1 hour at 95 °C. The reaction was stopped by cooling on ice and the samples were extracted with toluene. The absorbance of the organic phase was read at 520 nm, using toluene as a blank. Concentrations were calculated based on a standard curve prepared with known amounts of proline and expressed as μmol·g^−1^ DW.

### 4.7. MDA, Phenolics and Flavonoids Determination

Quantification of MDA, total phenolics, and flavonoids was performed in methanol extracts (80%, *v/v*, in water) obtained by grinding 0.05–0.10 g of fresh leaves in a mortar, shaking the samples on a rocker shaker overnight at room temperature, followed by centrifugation at 13,300× *g* for 15 min. MDA in the supernatants was quantified as previously described by Hodges et al. [99]. Each sample was mixed with 0.5% thiobarbituric acid (TBA) prepared in 20% trichloroacetic acid (TCA), or with 20% TCA without TBA for the controls, and then incubated at 95 °C for 15 min in a water bath. Reactions were stopped on ice and samples were centrifuged at 13,300 rpm for 10 min at 4 °C. The absorbance of the supernatants was measured at 532 nm. After subtracting the non-specific absorbance at 600 and 440 nm, the MDA concentration was calculated by applying the equations described by Hodges [99] based on the molar extinction coefficient of the MDA-TBA adduct at 532 nm (ε532 = 155 mM^−1^ cm^−1^).

The concentration of total phenolic compounds (TPCs) was determined according to the protocol of Blainski et al. [100], which is based on reaction with Folin–Ciocalteu reagent in the presence of NaHCO_3_. The reaction mixtures were incubated at room temperature in the dark for 90 min, and then the absorbance was recorded at 765 nm. TPC concentration was expressed as equivalents of the standard gallic acid (mg eq. GA·g^−1^ DW).

Total flavonoids (TFs) were determined by nitration of catechol groups with NaNO_2_, followed by the reaction with AlCl_3_ under alkaline conditions [101]. The absorbance of the samples was read at 510 nm, using catechin as the standard. TF concentration was expressed as equivalents of catechin (mg eq. C·g^−1^ DW).

### 4.8. Statistical Analysis

Data were analysed using Statgraphics Centurion XVI (Statgraphics Technologies, The Plains, VA, USA). The Levene test was applied to check whether analysis of variance (ANOVA) requirements were accomplished. Germination percentages were normalised by arcsine transformation prior to the analysis of variance. Significant differences be-tween treatments were tested by one-way analysis of variance (ANOVA) at the 95% confidence level, and post hoc comparisons were made using Tukey’s HSD test at *p* < 0.05. All mean values throughout the text are followed by SE. A multivariate analysis, r hierarchical clustering using Ward’s method, and Euclidean similarity index, were performed based on the means of all parameters measured in the plants, using the program called PAleontological STatistics (PAST) Version 4.09 Natural History Museum, University of Oslo, Norway [102].

## 5. Conclusions

*Erigeron bonariensis* and *E. sumatrensis* proved to be relatively tolerant to mild salinity and water stress, as plants were mostly affected by concentrations of 300 mM NaCl and higher. *E. bonariensis* seeds germinate generally in a higher percentage and faster, whereas during vegetative growth the two species respond similarly to stress. K^+^ homeostasis seems to be an essential mechanism in the stress tolerance of the two species, but an increased uptake was registered only in *E. bonariensis*. Proline concentration increased in all salt-stressed plants of *E. bonariensis*, but not in the water stressed plants. In *E. sumatrensis* proline increased only in plants from the 600 mM NaCl treatment, but constitutively high levels of proline were detected in this species. The data obtained suggest that the distribution areas of the two species may expand in the future due to their capacity to adapt to harsher environmental conditions triggered in many areas of the world by global warming.

## Figures and Tables

**Table 1:** Germination indices calculated for different treatments in the two *Erigeron* species: GI—germination index; SE—speed of emergence; CRG—coefficient of germination speed.

Species	Treatment	GI	SE	CRG
*E. bonariensis*	Control	69.0	31.3	27.9
	50 mM NaCl	9.8	42.9	26.4
	100 mM NaCl	6.2	41.7	23.1
	150 mM NaCl	4.4	14.3	18.9
	−0.21 MPa PEG	13.9	30.4	23.8
	−0.42 MPa PEG	13.8	35.6	22.8
	−0.63 MPa PEG	12.9	31.7	23.8
*E. sumatrensis*	Control	31.9	4.9	15.3
	50 mM NaCl	4.5	9.4	13.2
	100 mM NaCl	3.5	38.5	12.6
	150 mM NaCl	0.4	50.0	9.5
	−0.21 MPa PEG	6.0	16.1	17.1
	−0.42 MPa PEG	6.3	11.8	16.7
	−0.63 MPa PEG	4.7	3.3	14.7

**Table 2:** Two-way analysis of variance (ANOVA) of species, treatment and their interactions for the growth parameters considered.

Trait	Species	Treatment	Interaction	Residual
SL	9.05 **	23.54 ***	8.81	58.58
LA	6.22 *	8.05	8.98	76.72
Lno	37.84 ***	42.32 ***	6.53 ***	13.30
RFW	4.24 **	69.16 ***	2.74	23.84
LFW	7.00 ***	69.06 ***	3.53	20.39
RWC	4.24 **	69.16 ***	2.74	23.85
LWC	0.04	69.99 ***	11.93 ***	18.05

Numbers represent percentages of the sum of squares at the 5% confidence level. Abbreviations: SL: stem length; LA: leaf area; Lno: leaf number; RFW: root fresh weight; LFW: leaf fresh weight; RWC: root water content; LWC: leaf water content. Asterisks indicate the degree of significance: * *p* < 0.05, ** *p* < 0.01; *** *p* < 0.001, ns = not significant.

**Table 3:** Two-way analysis of variance (ANOVA) of species, treatment, and their interactions for the monovalent ions analysed.

Trait	Species	Treatment	Interaction	Residual
Na^+^_r_	19.00 ***	42.01 ***	11.53 **	29.29
Na^+^_l_	4.93 **	59.60 ***	2.73	32.70
Cl^−^_r_	8.86 **	25.01 ***	7.01	59.10
Cl^−^_l_	0.58	54.80 ***	3.72	40.90
K^+^_r_	23.60 ***	7.80 ***	41.87 ***	26.71
K^+^_l_	5.15	11.70	4.45	78.70

Numbers represent percentages of the sum of squares at the 5% confidence level. Abbreviations: Na^+^_r_: sodium in roots; Na^+^_l_: sodium in leaves; Cl^−^_r_: chlorine in roots; Cl^−^_l_: chlorine in leaves; K^+^_r_: potassium in roots; K^+^_l_: potassium in leaves. Asterisks indicate the degree of significance: ** *p* < 0.01; *** *p* < 0.001, ns = not significant.

**Table 4:** Two-way analysis of variance (ANOVA) of species, treatment and their interactions for the biochemical parameters analysed.

Trait	Species	Treatment	Interaction	Residual
Chl a	2.14	8.67	4.99	84.8
Chl b	0.17	6.33	3.31	90.17
Pro	23.65 ***	30.07 ***	15.91 ***	30.36
MDA	10.14	10.07	6.28	73.48
TPC	58.72 ***	11.11 **	4.28	25.87
TF	0.01	9.94	11.91	78.13

Numbers represent percentages of the sum of squares at the 5% confidence level. Abbreviations: Chl a: chlorophyll a; Chl b: chlorophyll b; Pro: proline; MDA: malondialdehyde; TPC: total phenolic compounds; TF: total flavonoids. Asterisks indicate the degree of significance: ** *p* < 0.01; *** *p* < 0.001, ns = not significant.

## Data Availability

Not applicable.
